# Supplementary material for: Towards in silico CLIP-seq: predicting protein-RNA interaction via sequence-to-signal learning
Source: Genome Biol. 2023 Aug 4;24:180. doi: 10.1186/s13059-023-03015-7 (PMC10403857; doi:10.1186/s13059-023-03015-7)
Supplement: Supplementary file 1 — Additional file 1. Includes additional text on the expected variance of replicates, additional figures for the RBPNet architecture, consensus motifs, mixing coefficient analysis and variant scoring analysis. [file 13059_2023_3015_MOESM1_ESM.pdf]

Supplementary Text

Variance of replicates

Assume that x and y are repeated measurements with (unknown) mean m and independent noise with variance v. We have an estimate of the mean $\widehat{m}$. We can calculate the expected squared derivation of the two replicates:

$$\mathbb{E}[(x-y)^2] = \mathbb{E}[(x-m-(y-m))^2] = 2v - 2\mathbb{E}[(x-m)(y-m)] = 2v \ ,$$

where the cross term disappears because the noise is assumed to be independent on the two replicates.

We can also calculate the squared difference between the estimate and a replicate:

$$\mathbb{E}[(x-\widehat{m})^2] = v + (m-\widehat{m})^2.$$

This results follows from the same type of reasoning as above. From these two results we can see that if our estimator is equal to the true mean: $\widehat{m} = m$ then:

$$\mathbb{E}[(x-y)^2] = 2\,\mathbb{E}[(x-\widehat{m})^2].$$

It follows that as the estimated mean $\widehat{m}$ approaches the true mean, the squared derivation of the two replicates exceeds the squared derivation of a replicate and $\widehat{m}$ by v.

Supplementary Figures

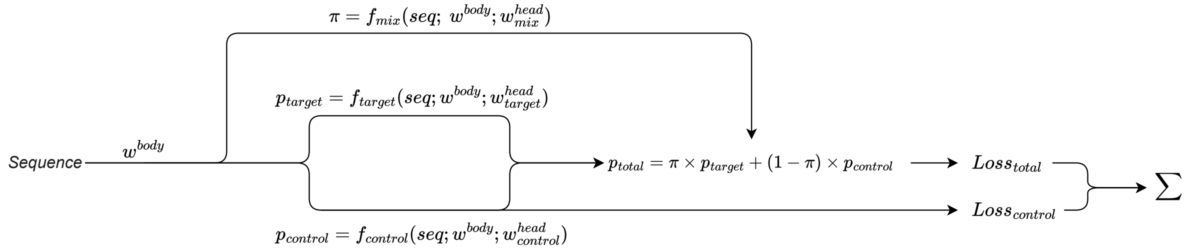

(a)

Figure S1: RBPNet forward pass (with bias correction). The feature representation of the input RNA sequence (parameterized by w^{body}) is used to compute the count distribution of the target (p_{target}) and control ($p_{control}$) tracks. The count distribution of the total track is then given by an additive mixture of target and control tracks, as well a mixing coefficient $\pi \in [0, 1]$. Given the observed counts of the CLIP and control experiment, losses are computed using the total and control tracks, respectively. The total loss is then given by the sum of the total and control track losses.

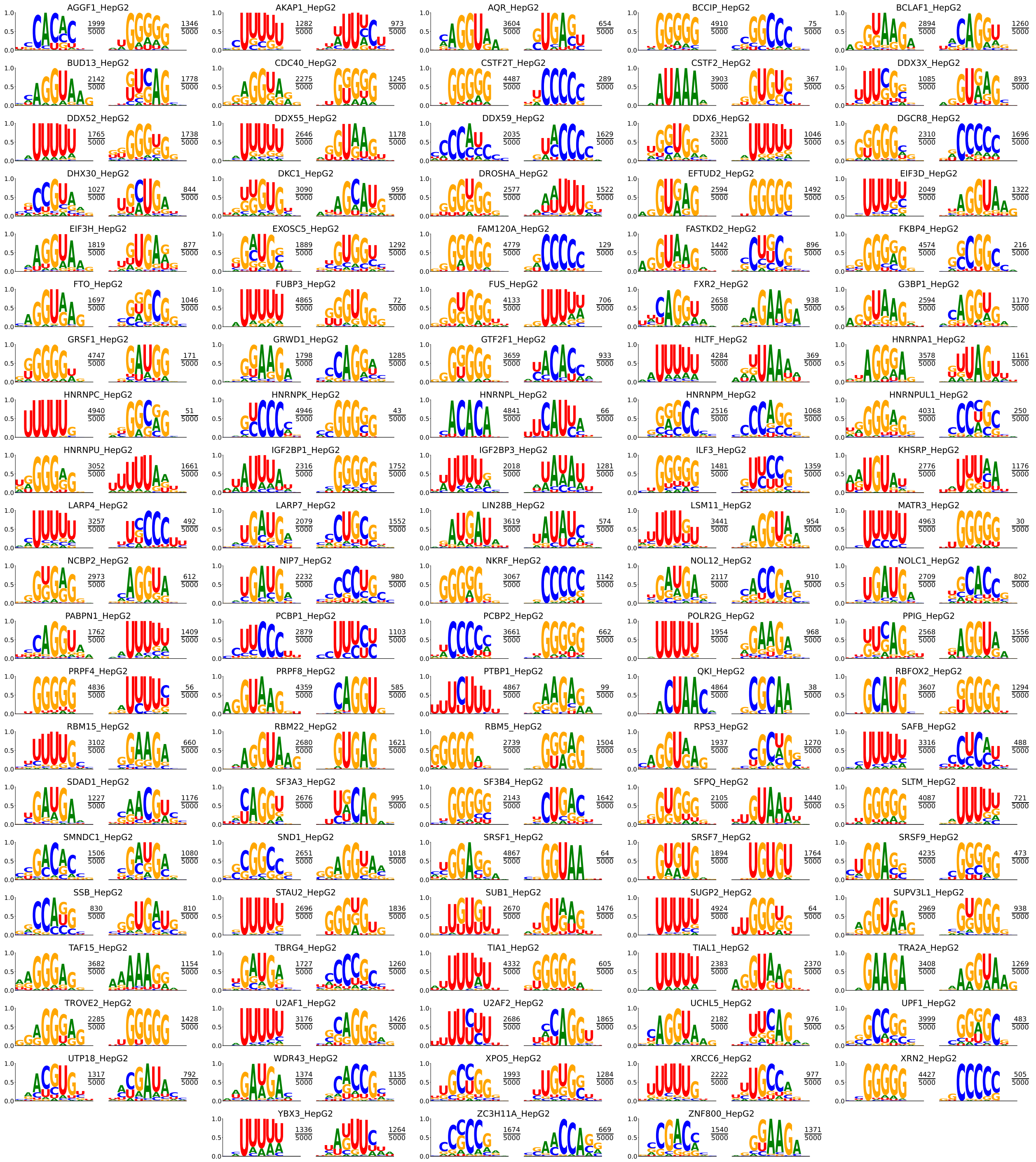

Figure S2: Consensus motifs with highest and second-highest 5-mer support for all 103 ENCODE HepG2 eCLIP experiments.

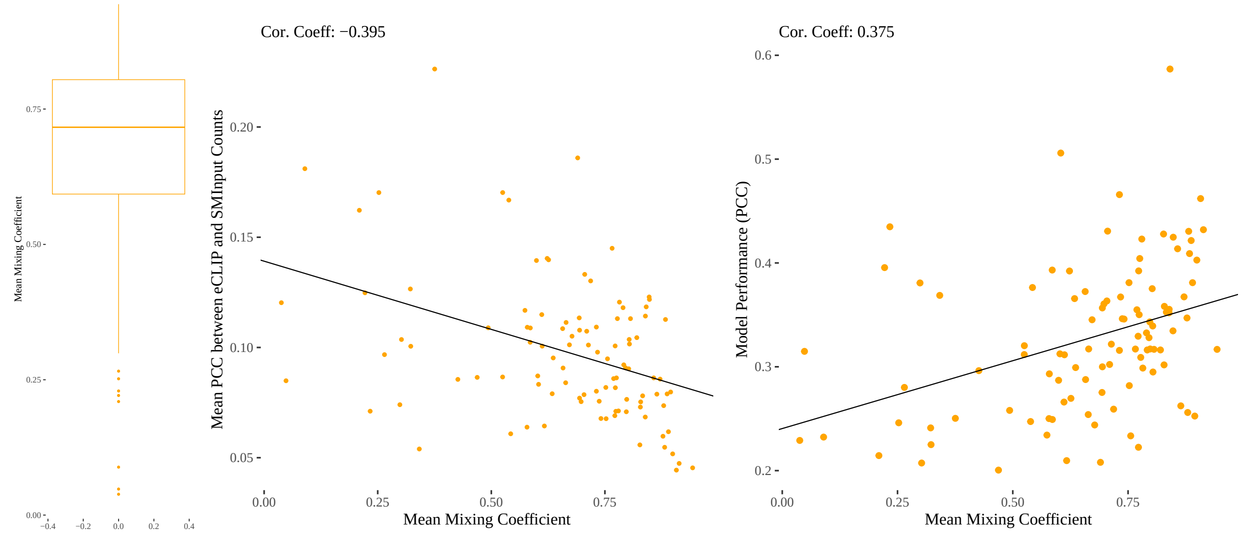

(a) (b) (c)

Figure S3: **A** Distribution of average the mixing coefficient on hold-out samples across 103 ENCODE HepG2 eCLIP experiments. **B** Correlation of the average mixing coefficient and the average similarity of eCLIP and paired control (SMInput), measured as PCC across hold-out samples. **C** Correlation of the average mixing coefficient and RBPNet performance, measured as PCC between predicted and observed count distribution of the total (eCLIP) track.

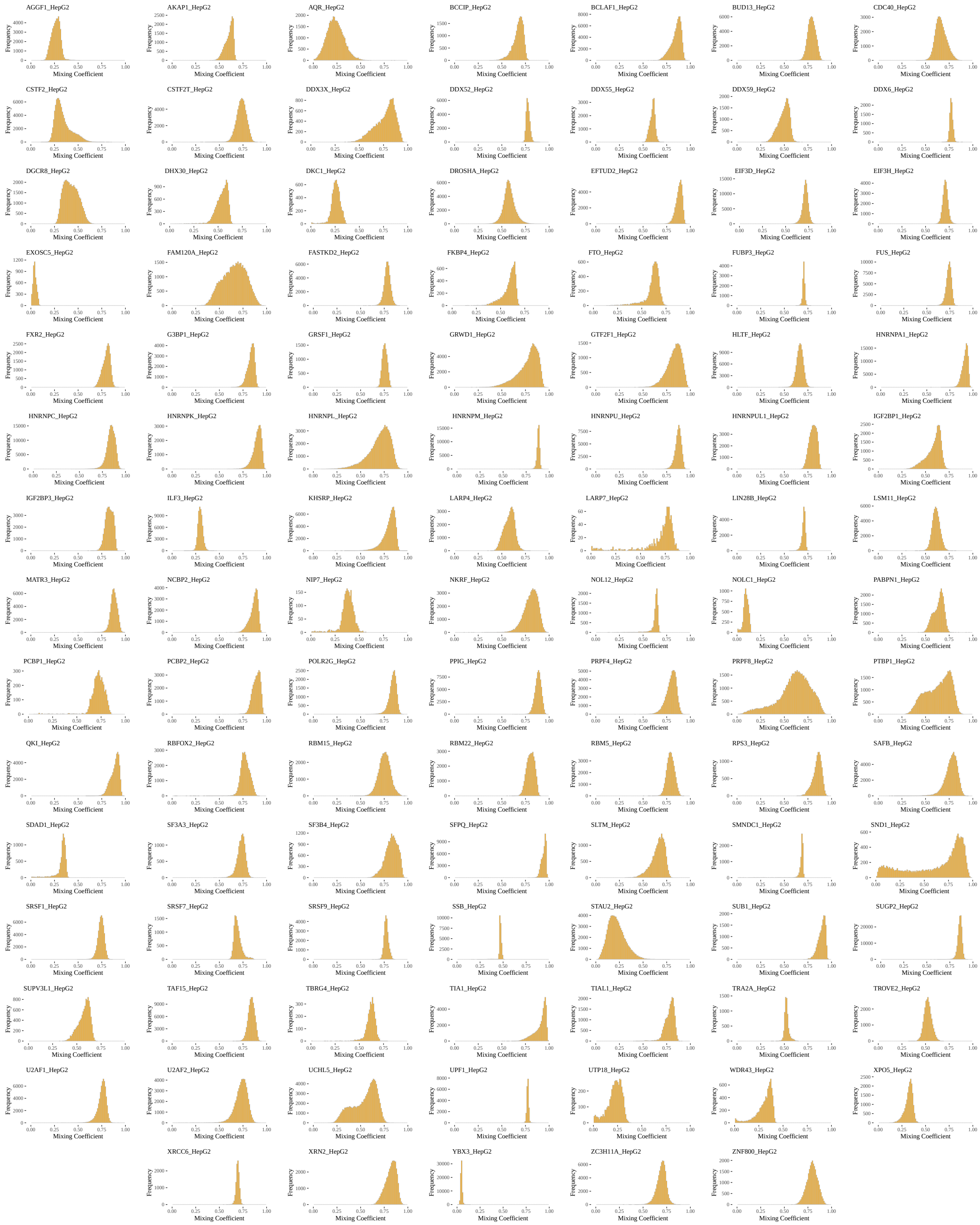

Figure S4: Distribution of predicted RBPNet mixing coefficients on hold-out samples for each eCLIP experiment.

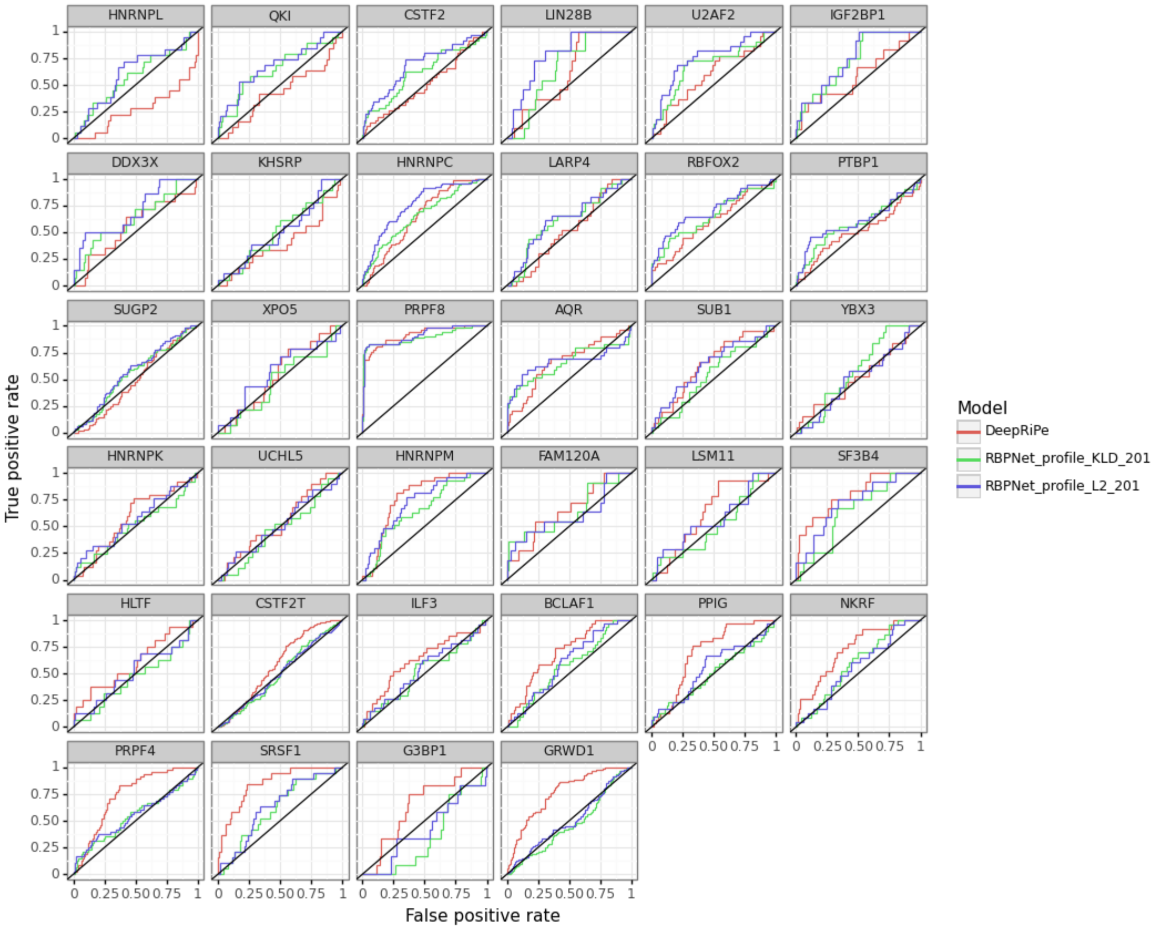

Figure S5: Protein-wise ROC curves for ASB vs. non-ASB classification of DeepRiPe and RBPNet. In case of RBPNet, reference and alternative allele predictions were compared using KL divergence and the L2-norm of the element-wise difference between the two vectors.

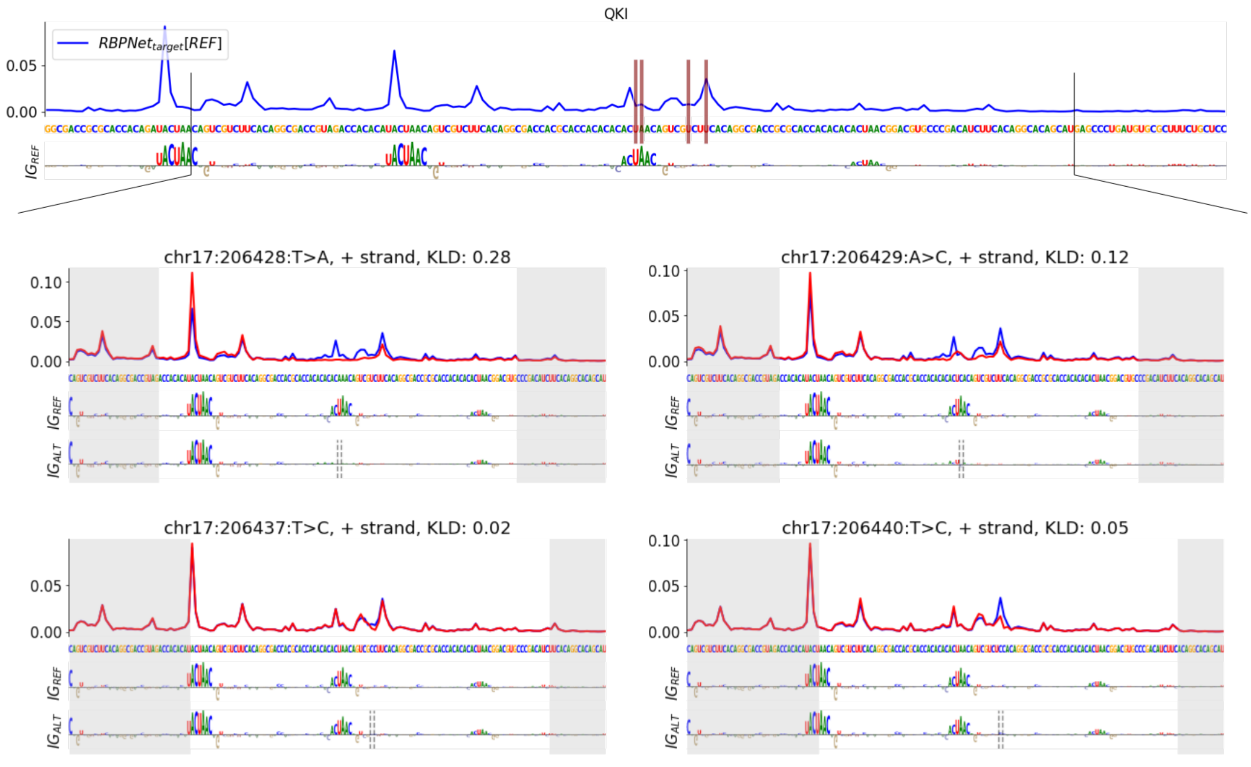

(a)

Figure S6: Variant impact predictions for the QKI model in a region harboring 4 distinct SNVs associated with ASB (Yang et al. [67]). The top plot shows the reference sequence with the corresponding attribution map. The location of the SNVs within the sequence is highlighted with red bars. The 4 SNVs are displayed separately, showing a comparison of the predicted profile with the reference (top) together with the attribution maps of the reference and the alternative allele (bottom). The location of the variant is highlighted with a dashed grey line in the attribution map of the alternative allele. The grey boxes represent the margins of the KLD scoring window centered around the variant. RBPNet predicts notable allele specific effects for only 2 out of the 4 candidate SNVs, which coincide with QKI binding motifs. These SNVs are associated with a loss of the binding motif in the attribution map and a change in the predicted signal.

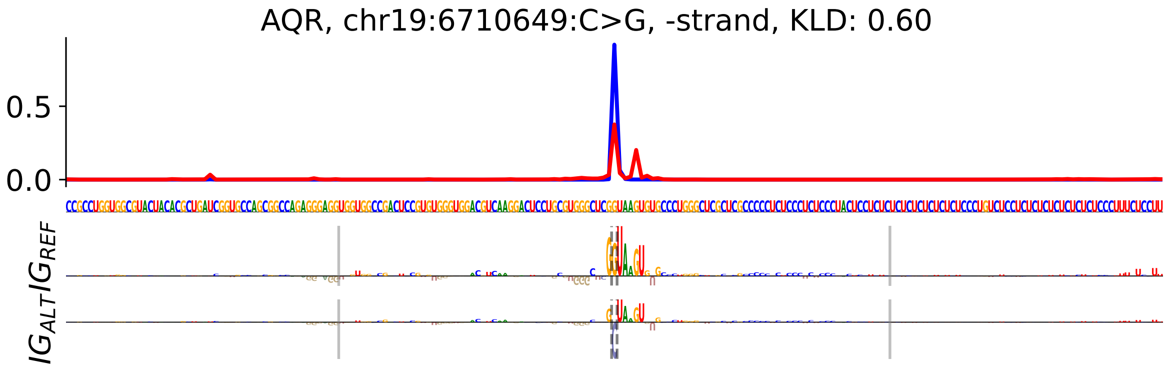

(a)

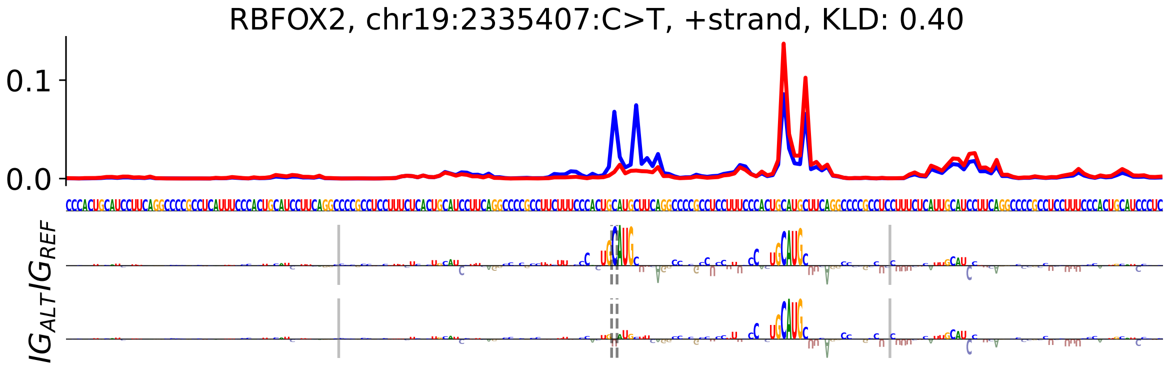

(b)

Figure S7: Example impact predictions of the most significant allele-specific binding (ASB) events identified by Yang et al. [67] for AQR (**A**) and RBFOX2 (**B**).

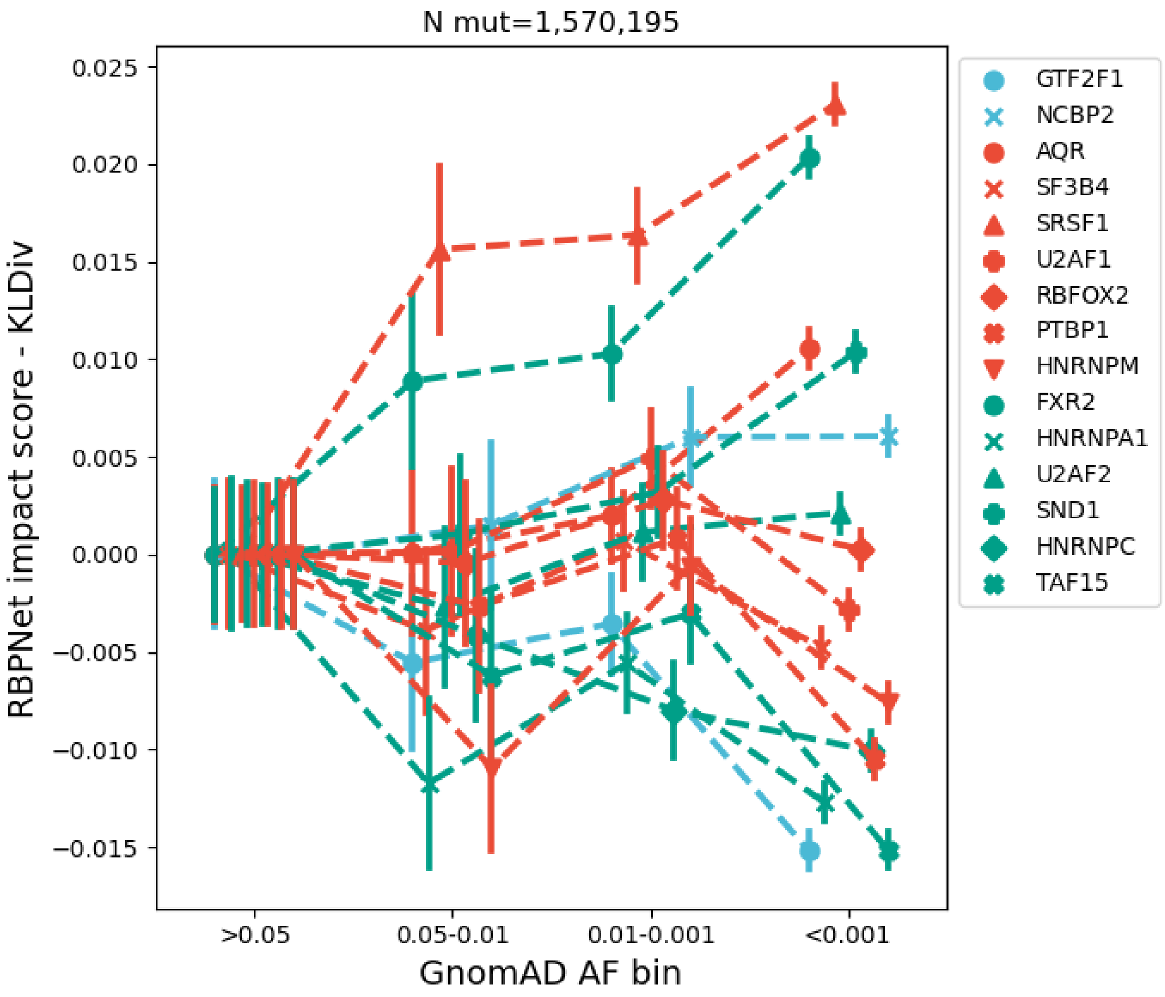

Figure S8: RBPNet mean variant impact score (measured in terms of KL divergence) as a function of allele frequency across gnomAD variants.

Supplementary Tables

Table S1: AP and auROC performance of RBPNet (target track) on 103 ENCODE eCLIP experiments from the HepG2 cell line.

	RBP_CELL	AP	auROC
1	AGGF1_HepG2	0.02	0.83
2	AKAP1_HepG2	0.05	0.88
3	AQR_HepG2	0.15	0.83
4	BCCIP_HepG2	0.08	0.95
5	BCLAF1_HepG2	0.10	0.94
6	BUD13_HepG2	0.09	0.92
7	CDC40_HepG2	0.05	0.87
8	CSTF2_HepG2	0.12	0.93
9	CSTF2T_HepG2	0.07	0.94
10	DDX3X_HepG2	0.09	0.95
11	DDX52_HepG2	0.09	0.87
12	DDX55_HepG2	0.07	0.89
13	DDX59_HepG2	0.05	0.87
14	DDX6_HepG2	0.00	0.89
15	DGCR8_HepG2	0.08	0.82
16	DHX30_HepG2	0.04	0.87
17	DKC1_HepG2	0.11	0.74
18	DROSHA_HepG2	0.04	0.86
19	EFTUD2_HepG2	0.04	0.84
20	EIF3D_HepG2	0.05	0.91
21	EIF3H_HepG2	0.04	0.92
22	EXOSC5_HepG2	0.01	0.64
23	FAM120A_HepG2	0.08	0.94
24	FASTKD2_HepG2	0.06	0.90
25	FKBP4_HepG2	0.17	0.92
26	FTO_HepG2	0.04	0.83
27	FUBP3_HepG2	0.10	0.93
28	FUS_HepG2	0.07	0.90
29	FXR2_HepG2	0.05	0.96
30	G3BP1_HepG2	0.03	0.90
31	GRSF1_HepG2	0.02	0.79
32	GRWD1_HepG2	0.10	0.95
33	GTF2F1_HepG2	0.10	0.90
34	HLTF_HepG2	0.04	0.89
35	HNRNPA1_HepG2	0.11	0.93
36	HNRNPC_HepG2	0.23	0.98
37	HNRNPK_HepG2	0.16	0.98
38	HNRNPL_HepG2	0.16	0.98
39	HNRNPM_HepG2	0.09	0.97
40	HNRNPU_HepG2	0.05	0.90
41	HNRNPUL1_HepG2	0.05	0.83
42	IGF2BP1_HepG2	0.07	0.94
43	IGF2BP3_HepG2	0.04	0.93
44	ILF3_HepG2	0.04	0.91
45	KHSRP_HepG2	0.07	0.89
46	LARP4_HepG2	0.07	0.88
47	LARP7_HepG2	0.20	0.79
48	LIN28B_HepG2	0.04	0.91
49	LSM11_HepG2	0.04	0.87
50	MATR3_HepG2	0.08	0.97
51	NCBP2_HepG2	0.09	0.91
52	NIP7_HepG2	0.09	0.84

	RBP_CELL	AP	auROC
53	NKRF_HepG2	0.05	0.89
54	NOL12_HepG2	0.15	0.81
55	NOLC1_HepG2	0.18	0.76
56	PABPN1_HepG2	0.05	0.89
57	PCBP1_HepG2	0.09	0.87
58	PCBP2_HepG2	0.14	0.97
59	POLR2G_HepG2	0.09	0.97
60	PPIG_HepG2	0.11	0.96
61	PRPF4_HepG2	0.11	0.95
62	PRPF8_HepG2	0.22	0.97
63	PTBP1_HepG2	0.25	0.93
64	QKI_HepG2	0.17	0.96
65	RBFOX2_HepG2	0.11	0.96
66	RBM15_HepG2	0.08	0.91
67	RBM22_HepG2	0.10	0.93
68	RBM5_HepG2	0.07	0.84
69	RPS3_HepG2	0.12	0.95
70	SAFB_HepG2	0.09	0.90
71	SDAD1_HepG2	0.08	0.68
72	SF3A3_HepG2	0.12	0.91
73	SF3B4_HepG2	0.12	0.96
74	SFPQ_HepG2	0.04	0.88
75	SLTM_HepG2	0.05	0.92
76	SMNDC1_HepG2	0.19	0.82
77	SND1_HepG2	0.12	0.96
78	SRSF1_HepG2	0.12	0.97
79	SRSF7_HepG2	0.10	0.93
80	SRSF9_HepG2	0.15	0.94
81	SSB_HepG2	0.01	0.58
82	STAU2_HepG2	0.07	0.91
83	SUB1_HepG2	0.04	0.88
84	SUGP2_HepG2	0.06	0.91
85	SUPV3L1_HepG2	0.09	0.74
86	TAF15_HepG2	0.07	0.89
87	TBRG4_HepG2	0.00	0.70
88	TIA1_HepG2	0.08	0.93
89	TIAL1_HepG2	0.10	0.95
90	TRA2A_HepG2	0.12	0.77
91	TROVE2_HepG2	0.10	0.86
92	U2AF1_HepG2	0.12	0.95
93	U2AF2_HepG2	0.16	0.98
94	UCHL5_HepG2	0.09	0.93
95	UPF1_HepG2	0.04	0.89
96	UTP18_HepG2	0.08	0.74
97	WDR43_HepG2	0.09	0.74
98	XPO5_HepG2	0.01	0.82
99	XRCC6_HepG2	0.04	0.88
100	XRN2_HepG2	0.09	0.92
101	YBX3_HepG2	0.02	0.82
102	ZC3H11A_HepG2	0.06	0.88
103	ZNF800_HepG2	0.09	0.95
